# Mapping the characteristics of network meta-analyses on drug therapy: A systematic review

**DOI:** 10.1371/journal.pone.0196644

**Published:** 2018-04-30

**Authors:** Fernanda S. Tonin, Laiza M. Steimbach, Antonio M. Mendes, Helena H. Borba, Roberto Pontarolo, Fernando Fernandez-Llimos

**Affiliations:** 1 Pharmaceutical Sciences Postgraduate Programme, Federal University of Parana, Curitiba, Parana, Brazil; 2 Department of Pharmacy, Federal University of Parana, Curitiba, Parana, Brazil; 3 Research Institute for Medicines (iMed.ULisboa), Universidade de Lisboa, Lisbon, Portugal; 4 Department of Social Pharmacy, Faculty of Pharmacy, Universidade de Lisboa, Lisbon, Portugal; McMaster University, CANADA

## Abstract

**Background:**

Network meta-analysis (NMA) is a new tool developed to overcome some limitations of pairwise meta-analyses. NMAs provide evidence on more than two comparators simultaneously. This study aimed to map the characteristics of the published NMAs on drug therapy comparisons.

**Methods:**

A systematic review of NMAs comparing pharmacological interventions was performed. Searches in Medline (PubMed) and Scopus along with manual searches were conducted. The main characteristics of NMAs were systematically collected: publication metadata, criteria for drug inclusion, statistical methods used, and elements reported. A methodological quality score with 25 key elements was created and applied to the included NMAs. To identify potential trends, the median of the publication year distribution was used as a cut-off.

**Results:**

The study identified 365 NMAs published from 2003 to 2016 in more than 30 countries. Randomised controlled trials were the primary source of data, with only 5% including observational studies, and 230 NMAs used a placebo as a comparator. Less than 15% of NMAs were registered in PROSPERO or a similar system. One third of studies followed PRISMA and less than 9% Cochrane recommendations. Around 30% presented full-search strategies of the systematic review, and 146 NMAs stated the selection criteria for drug inclusion. Over 75% of NMAs presented network plots, but only half described their geometry. Statistical parameters (model fit, inconsistency, convergence) were properly reported by one third of NMAs. Although 216 studies exhibited supplemental material, no data set of primary studies was available. The methodological quality score (mean 13·9; SD 3·8) presented a slightly positive trend over the years.

**Conclusion:**

The map of the published NMAs emphasises the potential of this tool to gather evidence in healthcare, but it also identified some weaknesses, especially in the report, which limits its transparency and reproducibility.

## Introduction

Traditional pairwise meta-analyses produced a step forward in evidence-based selection between therapeutic alternatives. However, the lack of a complete set of head-to-head clinical trials limits the evidence in many areas [1,2]. This situation is especially relevant in highly innovative therapeutic classes, in which trials comparing two drugs require large sample sizes and financial resources [1,3,4]. In addition, traditional pairwise meta-analyses are restricted to compare only two treatments at a time [5–8].

The indirect comparison method proposed by Bucher et al [9] provided a potential solution for treatments that have not been directly compared before. However, this model can only be applied to data generated from trials with two arms and with a common comparator, allowing the indirect comparison of three treatments (A vs. B; B vs. C) [10,11]. Thereafter, Lumley [12] and Lu and Ades [13] improved indirect treatment comparison techniques, involving more than one common comparator (the linking treatment) and creating NMA, also called mixed or multiple treatment comparison meta-analysis. NMA allows to simultaneously combine both direct and indirect results from all studies’ arms into a single pooled effect, which strengthens results and provides a broader picture of all treatments in the same model [14–17]. Moreover, NMAs calculate the probability for each treatment to be the best (or worst) for a specific outcome by creating probability rank orders or rankograms (graphical methods), which are useful for the decision-making process [11,18].

Over the last several years, NMA has matured as a technique, with models available for all types of raw data, producing different pooled effect measures, using both frequentist and Bayesian frameworks with different approaches (i.e. contrast-based or arm-based) and software packages available [19–26]. However, initial analyses of NMAs reported some gaps in the use of this new technique [19,27–29]. Thus, our aim was to map the characteristics of all the NMAs published, including drug therapy comparisons.

## Material and methods

### Search and eligibility criteria

A systematic review was performed according to PRISMA (Preferred Reporting Items for Systematic Reviews and Meta-Analyses) and Cochrane Collaboration recommendations [30,31]. Two reviewers performed all the steps individually, and discrepancies were decided by a third author.

We searched for articles reporting NMAs comparing drug therapy alternatives in PubMed and Scopus without time or language limits (last updated in March 2016). A manual search in the reference lists of included studies was performed, and grey literature was also searched in Google and Google Scholar. The complete search strategies are presented as supporting information (S1 Table).

We included studies using NMAs—also referred to as multiple or mixed treatment comparisons, mixed treatment meta-analysis, or indirect meta-analysis—to compare any drug therapy intervention (defined as a pharmacological intervention including an active substance) alone or in combination with other pharmacological intervention, regardless of regimen or dosage. We considered any type of network (with open or closed loops) of experimental, quasi-experimental, or observational trials that assessed at least three or more treatments, comparing head to head or against placebo/no control in patients (no restriction of gender, age, or clinical/medical condition). Non-NMAs, study protocols, studies reporting data only on non-pharmacological interventions, and articles written in non-Roman characters were excluded during screening (title and abstract reading) and full-text article eligibility steps.

### Data extraction and analyses

We used a standardised data collection form to extract data on: (i) the studies’ general characteristics, such as author names, countries of affiliation, journal impact factor (as reported on journal citation reports), publication year, sample size (number of included trials and population), type of included studies, and patients’ clinical conditions; (ii) methods used in the systematic review (included databases, description of complete search strategies, reports on manual search, grey literature searches, recommendation compliance, register [PRISMA–Preferred Reporting Items for Systematic Reviews and Meta-Analyses statement, Cochrane Recommendations, PROSPERO–International prospective register of systematic reviews]) and the studies’ quality assessment using validated methods (i.e. Jadad Score or Cochrane Risk of Bias Tool); (iii) description of statistical analyses (frequentist, Bayesian, or both), statistical model (random, fixed, or both), statistical approaches (i.e. contrast-based or arm-based), additional analyses (i.e. subgroup, sensitivity, trial-level outlier detection, or meta-regression analyses), inconsistency analyses, model fit and model convergence, and computer software used for calculations; (iv) report of results (i.e. supplementary material; data on direct, indirect, or mixed evidence; presence of network plot; description of network geometry; and presence of rank orders); (v) conflict of interest and funding source declarations.

A methodological quality score with 25 key elements for the performance and reporting of systematic reviews and NMAs was applied. The construction of this preliminary tool was based on PRISMA-NMA statement, and considered the Bayesian approach for conduct NMAs due to its flexibility and interpretability. The main elements of conduct and reporting the systematic review process and the statistical analyses of NMAs were incorporated in this preliminary tool, considering both internal validity and reporting quality items. The complete quality score description is presented as supporting information (see S2 Table). Potential correlation of the methodological quality score was tested with (i) the year of publication of the NMA, (ii) the impact factor of the journal in which the NMA was published, and (iii) the area of the clinical condition evaluated by the NMA (e.g. cardiovascular diseases, metabolic disorders, respiratory diseases).

### Statistical analyses

To evaluate potential time trends, the median of the publication year distribution was used as a cut-off. The normality of the variables was assessed through the Kolmogorov–Smirnov and Shapiro–Wilk tests. Continuous variables with non-normal distribution were reported as median and interquartile range (IQR), and the Wilcoxon–Mann–Whitney test was used for within-group comparisons. Categorical variables were compared using the chi-square test for univariate comparisons and reported as absolute and relative frequencies. The methodological quality score (normally distributed) was correlated with the year of publication and impact factor through the Pearson test. ANOVA was used to associate the methodological quality score and clinical conditions. All analyses were conducted in IBM SPSS Statistics v. 24.0 (Armonk, NY: IBM Corp.), and probabilities below the 5% level were considered statistically significant.

## Results

**Fig 1:**
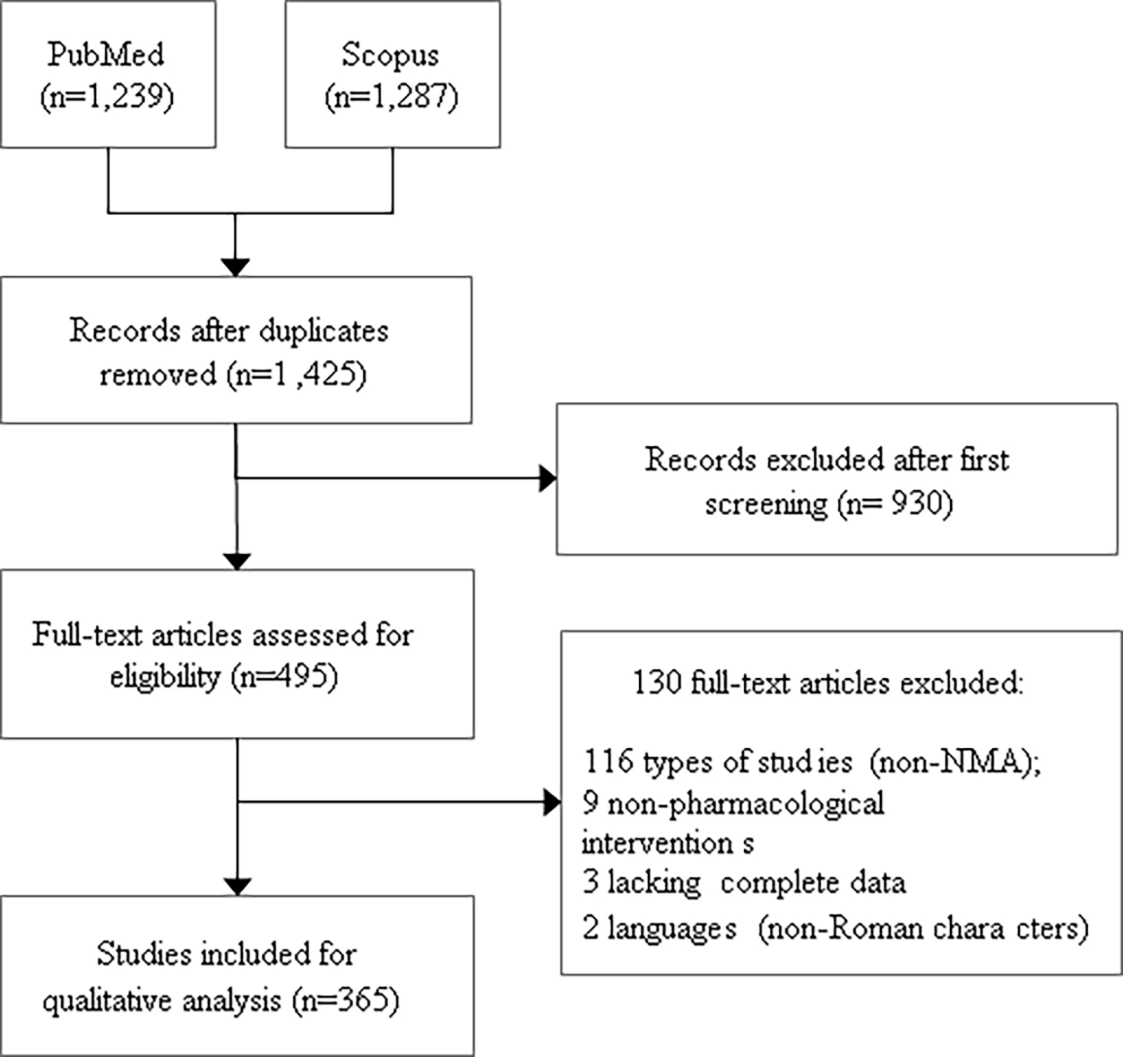
Flowchart of included network meta-analyses.

After the systematic search, a total of 1,425 articles were retrieved from PubMed and Scopus. During the screening process, 930 articles were considered irrelevant, and another 130 articles were excluded during the full-text appraisal, resulting in 365 NMAs for data extraction (Fig 1). For the complete raw data of the included NMA see OSF platform (DOI: 10.17605/OSF.IO/GVQXT).

**Fig 2:**
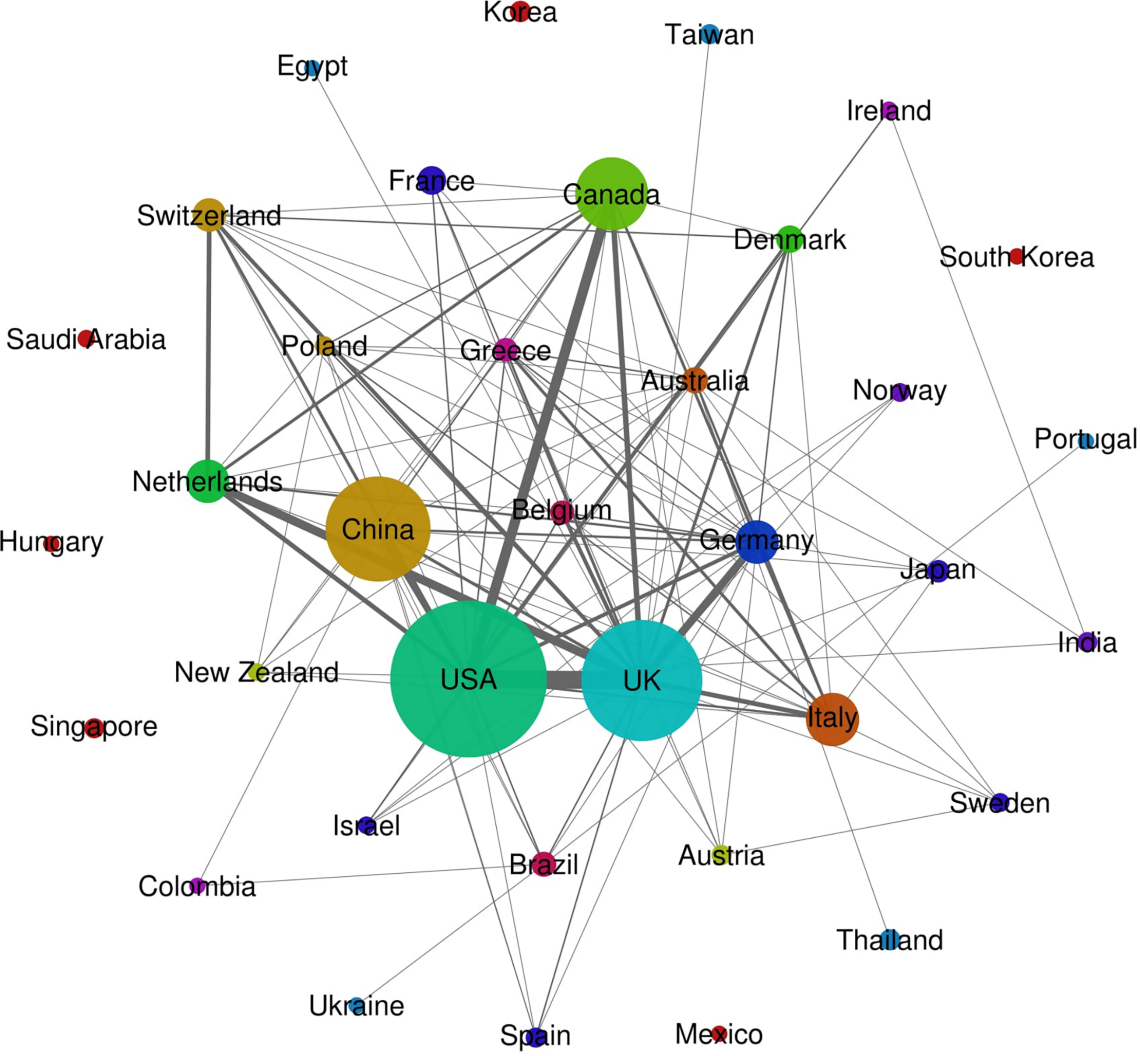
Graph of collaborative publications of NMAs. Countries are presented as nodes. Nodes sizes are proportional to the number of NMAs publications by country. Lines thickness are proportional to the number of NMAs publications between countries publishing in collaboration.

These 365 articles were published between 2003 and 2016, with a median in 2014 and an inflection point in 2010 (see Table 1). Most studies (n = 265; 72.6%) were produced in only one country: the United States (n = 62), China (n = 57), the United Kingdom (n = 33), Canada (n = 27), and Italy (n = 20). International collaboration among authors did not statistically differ before and after 2014, accounting for 24.7% of all studies published (Table 1). Switzerland, the Netherlands, and Germany were the most collaborative countries, with 80%, 78.3%, and 69.6% of articles published in collaboration, respectively. The final map of NMA publications (Fig 2) shows that the United States published more NMAs (n = 115), followed by the United Kingdom (n = 86) and China (n = 73). The medical conditions evaluated were cardiovascular diseases (n = 98), oncologic disorders (n = 50), autoimmune disorders (n = 39), mental health disorders (n = 32), infectious diseases (n = 32), respiratory diseases (n = 27), musculoskeletal disorders (n = 10), pain (n = 7), gastrointestinal injuries (n = 6), and other health disorders (n = 64), which included diseases of different systems (skin, eye, endocrine, genitourinary). The 365 NMAs were published in 204 different journals, but a decline in impact factor of the journals in which NMAs were published was observed. Prior to 2014, the mean impact factor of journals publishing NMAs was 6.214; after 2014, the mean impact factor was 4.701 (Table 1).

**Table 1:** Descriptive characteristics of articles reporting network meta-analyses.

	No. of networks reporting data	Total	Publication date		p value
			Prior to 2014	2014 or later	
International collaboration: N (%)	365	100 (27.4%)	39 (31.7%)	61 (25.2%)	0.117**
Journal impact factor (2015): mean (SD)	365	5.210 (6.445)	6.214 (7.520)	4.701 (5.775)	0.080*
Reports PROSPERO register: N (%)	365	53 (14.5%)	10 (8.1%)	43 (17.8%)	0.013**
Reports following PRISMA statement: N (%)	365	116 (31.8%)	19 (15.4%)	97 (40.1%)	<0.001**
Reports following Cochrane recommendations: N (%)	365	32 (8.8%)	12 (9.8%)	20 (8.3%)	0.634**
Criteria to select drugs in the network: N (%)	365				0.372**
No criterion		132 (36.2%)	46 (37.4%)	86 (35.5%)	
Non-objective criterion		87 (23.8%)	24 (19.5%)	63 (26.0%)	
Objective criterion		146 (40.0%)	53 (43.1%)	93 (38.4%)	
No. databases searched: median (range)	342	3.0 (1–19) IQR = 1	3.0 (1–10) IQR = 1	3.0 (1–19) IQR = 1	0.229*
No. studies included: median (range)	360	21 (3–450) IQR = 24	20 (3–218) IQR = 26	21 (3–450) IQR = 24	0.706*
No. included patients: median (range)	246	7625.50 (162–8977.48) IQR = 25177	10894.50 (162–3241.68) IQR = 31986	6852.00 (336–8977.48) IQR = 24390	0.019*
Provides complete search strategy: N (%)	365	108 (29.6%)	34 (27.6%)	74 (30.6%)	0.561**
Performs manual search: N (%)	365	268 (73.4%)	80 (65.0%)	188 (77.7%)	0.010**
Performs grey literature search: N (%)	365	176 (48.2%)	46 (37.4%)	130 (53.7%)	0.004**
Performs study quality assessment: N (%)	365	193 (52.9%)	51 (41.5%)	142 (58.7%)	0.002**
Provides supplemental material: N (%)	365	216 (59.2%)	63 (51.2%)	153 (63.2%)	0.027**
Reports conflicts of interest: N (%)	365				<0.001**
Not mentioned		39 (10.7%)	28 (22.8%)	11 (4.5%)	
Has conflicts		161 (44.1%)	52 (42.3%)	109 (45.0%)	
Has no conflicts		165 (45.2%)	43 (35.0%)	122 (50.4%)	
Reports financial support: N (%)	365				0.836**
Not mentioned		48 (13.2%)	18 (14.6%)	30 (12.4%)	
External support		202 (55.3%)	67 (54.5%)	135 (55.8%)	
No support		115 (31.5%)	38 (30.9%)	77 (31.8%)	

* Mann–Whitney

** chi-square

A protocol registration for the systematic review (i.e. PROSPERO) was provided by 53 studies (14.5%), and 31.8% studies (n = 116) stated complying PRISMA guideline. Both parameters significantly increased after 2014 (p values 0.013 and <0.001, respectively). Cochrane recommendations were followed only by 32 studies, of which 20 were published after 2014. Less than half of the articles (n = 146; 40%) reported objective criteria for the selection of drugs or classes included in the NMA, whereas 87 articles (23.8%) provided non-objective reasons (e.g. ‘most commonly used drugs’, ‘frequent treatments’, ‘currently employed drugs’). Studies occasionally provided complete search strategies (29.6%), with no significant differences before and after 2014 (p = 0.561).

The median number of databases used for the electronic searches was three (IQR = 1). The vast majority of the articles (342; 93.7%) detailed the databases used, with the following being the most frequent: PubMed/MEDLINE (92.9%), Cochrane Library (78.4%), Scopus/Embase (77.3%), Clinicaltrials.gov (17.0%), Web of Science (10.4%), CINAHL (6.3%), Health Technology Assessment (5.5%), and International Pharmaceutical Abstracts (1.9%). Manual searches and grey literature searches were conducted by 73.4% and 48.2% of studies, respectively. These two indicators along with the supply of online supplementary material (provided by 216 articles) have improved after 2014 (p values of 0.010, 0.004, and 0.027, respectively). The majority of NMAs (94.2%) included only randomised controlled trials, with the remaining 5.2% including also non-randomised or quasi-experimental trials or observational studies. Only two (0.5%) NMAs were restricted to observational studies. The median number of primary studies included in the networks (n = 21) remained similar before and after 2014 (p = 0.706). However, the median number of patients significantly decreased after 2014 (p = 0.019). Methodological quality assessment of primary studies was performed in 193 articles using the Jadad Score or the Cochrane Risk of Bias Tool. Over the years, more authors declared not to have conflicts of interest or did not mention any in their articles. More than 55% of studies received external financial support (Table 1).

As part of the NMA analyses, a network plot was provided by 287 articles for at least one assessed outcome (Table 2). The median number of nodes in the networks was 7.0 (IQR = 6), ranging from 3 to 71, with a statistically significant increase after 2014 (p value <0.001) of articles describing the geometry of the network (e.g. node sizes, line widths, proportion of trials and arms), as well as presenting rank order analyses of which intervention could be the best or worst for the clinical condition under evaluation (p value <0.001). A placebo was used as a comparator in 230 NMAs (63%). The statistical model used was described in 315 studies (86.3%), with the Bayesian model (n = 297) the most prevalent. The frequentist model was used in 15 articles, and both models were conducted by three studies. The statistical method was reported by 349 NMAs, with the random method (62.5%) the most common, and 33.8% of networks were built with both fixed and random methods. Only 3% of networks used only the fixed effect method. As expected, 91.8% of studies (n = 335) presented their main results as mixed treatment evidence, accounting for direct and indirect comparisons in one single effect (e.g. matrix of results, tables of data). Moreover, 52.9% of studies reported results for direct comparison and 12.1% for indirect comparisons individually. The software used was stated in 345 studies, with WinBUGS (57.5%), Stata (27.9%), R (23.8%), and Addis (6.0%) the most frequent. Supplementary analyses such as subgroup, sensitivity, and meta-regression analyses between included primary studies were conducted in about 60% of NMAs, and their prevalence was similar before and after 2014 (Table 2). The statistical approach (i.e. contrast-based or arm-based) was mentioned by 20% of studies (n = 73) and only 5.2% of NMA (n = 19) referred to be multivariate meta-analyses. The detection of outlying trials in the network was performed in only 26 studies (7.1%). However, there has been an increase of articles reporting network parameters such as inconsistency of direct and indirect evidence (p = 0.002), model fit (p = 0.333), and convergence (p = 0.004) in recent years.

**Table 2:** Characteristics of network meta-analyses.

	No. of networks reporting data	Total	Publication date		p value
			Prior to 2014	2014 or later	
Presents the network plot: N (%)	365	287 (78.6%)	92 (74.8%)	195 (80.6%)	0.203*
No. nodes: median (range)	287	7.0 (3–71); IQR = 6	7.0 (3–51) IQR = 4	7.0 (3–71) IQR = 5	0.191*
Describes the network geometry: N (%)	365	200 (54.8%)	50 (40.7%)	150 (62.0%)	<0.001*
Performs subgroup analyses: N (%)	365	50 (13.7)	21 (17.1%)	29 (12.0%)	0.181*
Performs sensitivity analyses: N (%)	365	207 (56.7%)	64 (52.0%)	143 (59.1%)	0.198*
Performs meta-regression: N (%)	365	59 (16.2%)	23 (18.7%)	36 (14.9%)	0.348*
Performs inconsistency analyses: N (%)	365	169 (46.3%)	43 (35.0%)	126 (52.1%)	0.002*
Performs model fit analyses: N (%)	365	119 (32.6%)	36 (29.3%)	83 (34.3%)	0.333*
Performs convergence analyses: N (%)	365	79 (21.6%)	16 (13.0%)	63 (26.0%)	0.004*
Presents rank order analysis: N (%)	365	216 (59.2%)	55 (44.7%)	161 (66.5%)	<0.001*

* Chi-square test.

**Fig 3:**
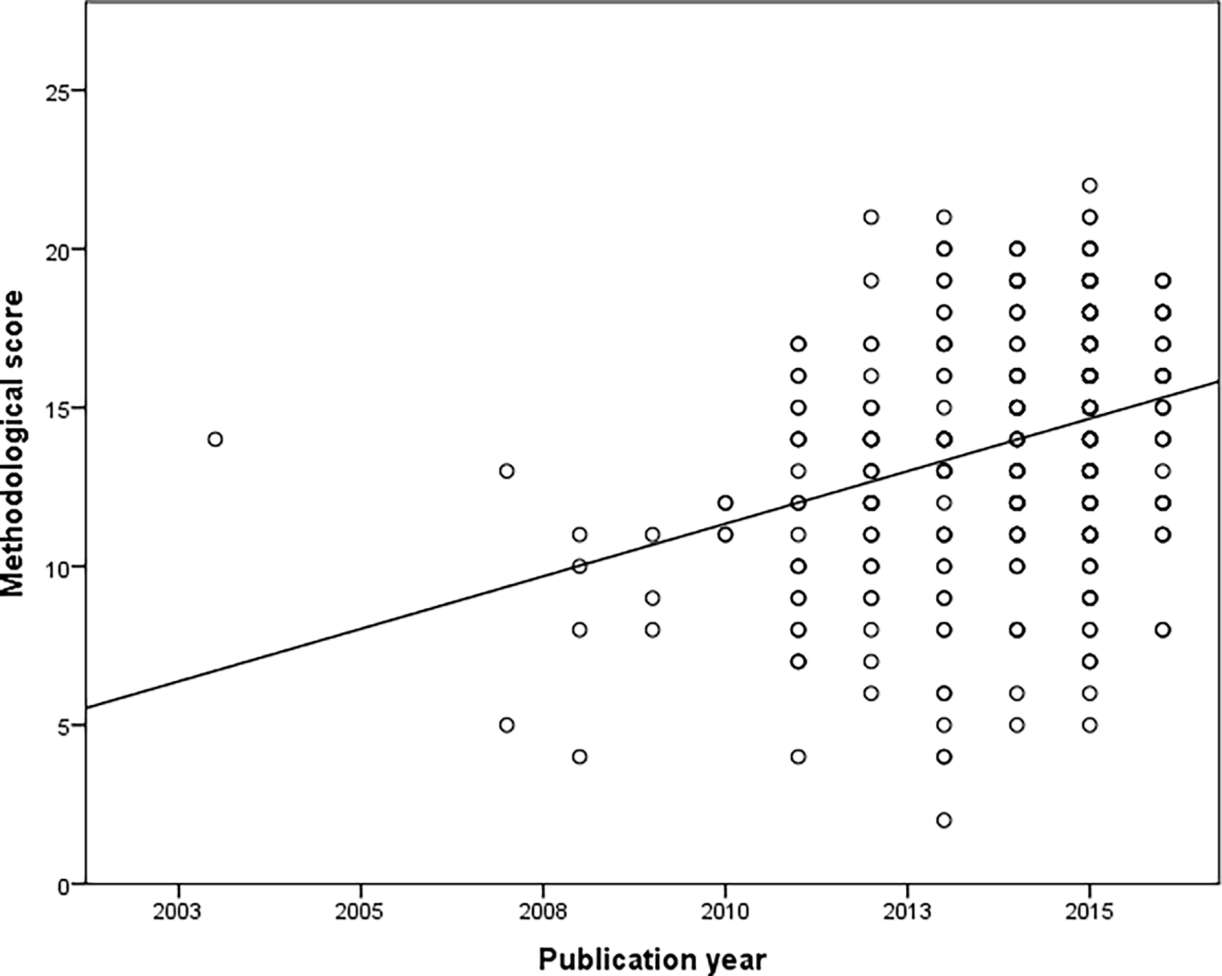
Correlation of methodological scores obtained by the NMA and the year of publication.

Overall, the 365 NMAs obtained a mean methodological quality score (considering items of internal validity and reporting quality) of 13.9 (SD = 3.8), ranging from 2 to 22. Before 2014, a mean of nine (SD 2.1) parameters was properly reported by at least half of studies (>50%), whereas after 2014, this number increased to 13 (SD 1.2) parameters. Reporting drug selection criteria as well as providing supplemental material have increased since 2012, whereas descriptions of NMAs’ geometry and rank order started increasing in 2013. However, parameters such as PROSPERO registration, PRISMA/Cochrane recommendation follow-up, and some statistical model descriptions are still poorly reported by authors (Table 3). A correlation was found for the year of publication of the NMA and the methodological quality score (r = 0.315) (Fig 3), whereas a slight correlation between the impact factor and the quality score was found (r = 0.172). No association was found between the quality score and the medical conditions of the NMAs (p = 0.437).

**Table 3:** Percentages of NMA studies reporting methodological parameters over the years.

Methodological Parameters	2003	2007	2008	2009	2010	2011	2012	2013	2014	2015	2016
PROSPERO register	0.00	0.00	0.00	0.00	0.00	0.00	0.11	0.13	0.22	0.17	0.15
PRISMA recommendations	0.00	0.00	0.00	0.00	0.00	0.12	0.20	0.19	0.28	0.44	0.50
Cochrane recommendations	0.00	0.00	0.00	0.00	0.00	0.12	0.09	0.13	0.06	0.09	0.09
Drug selection criteria	0.00	0.00	0.50	0.34	0.25	0.50	0.43	0.54	0.57	0.68	0.68
Search terms	1.00	0.50	0.25	0.00	0.75	0.69	0.66	0.69	0.68	0.58	0.59
Search strategies	0.00	0.00	0.00	0.00	0.25	0.42	0.17	0.33	0.34	0.27	0.38
More than two databases	1.00	1.00	0.75	0.33	1.00	0.65	0.74	0.88	0.87	0.87	0.88
Manual searches	0.00	0.50	0.50	0.33	0.75	0.62	0.74	0.65	0.77	0.81	0.68
Grey literature searches	0.00	0.50	0.50	0.00	0.25	0.34	0.54	0.27	0.49	0.60	0.35
Jadad/Cochrane bias evaluation	0.00	0.00	0.00	0.00	0.50	0.31	0.45	0.50	0.45	0.64	0.97
Supplemental material	0.00	0.50	0.25	0.33	0.50	0.58	0.49	0.54	0.67	0.61	0.54
Provides statistical analyses	0.00	0.50	0.75	0.33	0.75	0.77	0.80	0.81	0.86	0.92	0.94
Provides effect size measures	1.00	1.00	1.00	1.00	1.00	0.96	1.00	0.98	1.00	0.99	1.00
Provides statistical method	1.00	0.50	0.75	1.00	1.00	1.00	1.00	1.00	1.00	1.00	1.00
Additional statistical analyses	0.00	1.00	0.50	0.33	0.75	0.58	0.60	0.60	0.55	0.62	0.71
Software employed	1.00	0.50	1.00	1.00	1.00	1.00	0.91	0.92	0.96	0.94	0.97
Provides inconsistency	0.00	0.50	0.00	0.33	0.25	0.15	0.40	0.44	0.48	0.54	0.53
Provides model fit	0.00	0.00	0.00	0.33	0.00	0.31	0.34	0.31	0.34	0.34	0.35
Provides convergence	0.00	0.00	0.00	0.00	0.00	0.00	0.00	0.33	0.29	0.24	0.29
Provides results from NMA	0.00	1.00	0.75	1.00	0.75	0.88	0.94	0.85	0.91	0.94	0.97
Provides NMA plot	1.00	0.50	0.75	0.67	0.75	0.73	0.71	0.79	0.70	0.86	0.82
Provides NMA geometry	0.00	0.50	0.75	0.33	0.25	0.34	0.43	0.40	0.40	0.69	0.76
Provides rank order	0.00	0.00	0.50	0.67	0.50	0.42	0.37	0.50	0.65	0.64	0.79
COI (declared or none)	1.00	1.00	0.25	0.67	0.75	0.77	0.83	0.77	0.94	0.98	0.88
Financial support (declared or none)	1.00	1.00	0.50	1.00	1.00	0.85	0.91	0.81	0.91	0.88	0.76

Note: Studies were grouped by year. Percentages of studies properly reporting each parameter are represented. Percentages higher than 50% are colored green; the opposite 50% are red.

## Discussion

We identified a rapid increase in the publication of NMAs as a valid method to compare pharmacological treatments during the 2010s. Similar growth was previously reported for pairwise meta-analyses, whereas the annual publications increased more than 20-fold between 1994 (n = 386) and 2014 (n = 8203) [32–34]. The growing interest in NMAs is evident by more than 50% of NMAs published since 2014 by authors from more than 30 countries in more than 200 journals. Scientific production follows a geographical distribution associated with the number of researchers, available technology, country science funding, and international collaboration [33–35]. A study about the global production of pairwise meta-analyses (n = 736) published by 3,178 authors from 51 countries reported that developed countries such as the USA, the UK, and Canada were the greatest producers [36]. Similar results were found in our study, but with the emergence of new countries such as China and Italy, which may change the future publication patterns of NMAs [37,38]. New countries may enter, probably because NMAs are a valid, cheap, and quick alternative to support pricing and marketing approval decisions, especially in the absence of direct comparisons [27,39,40]. The increasing rate of NMA publications may also have caused the decrease of the impact factor of the journals publishing NMAs. The very low slope of the correlation between impact factor and NMAs’ methodological quality score suggests virtually no association. It seems that when NMAs were an innovative statistical tool, journals with the highest impact factor were more interested in this technique. However, with the increase of NMA production more journals became interested, including those with lower impact factors.

The quality of reports about methodological aspects in both systematic reviews and NMAs has also significantly improved over the years. Similarly to systematic reviews [31,41,42], more NMAs have performed manual and grey literature searches. We also found that more NMAs followed the PRISMA statement and provided a PROSPERO registration number. However, although Cochrane guidelines were available since 1994, few NMAs claimed to pursue these recommendations. Though authors have searched in more than two electronic databases, as recommended [31,43], only one third provided the complete search strategies, as similarly reported in a study on the systematic review process of NMAs [44]. As expected, PubMed/Medline was the most commonly used database for electronic searches, perhaps because of the expanded coverage of biomedicine and health sciences [45] and its free access. On the other hand, Web of Science was used only by one in ten NMAs. The highly restrictive process for journal indexing performed in the Web of Science, which is alleged as a strength to calculate the impact factor [46–48], may also be the reason why this database is considered useless in about 90% of NMA searches.

Probably one of the most important weaknesses of many NMAs is the lack of inclusion and exclusion criteria of molecules [49–51]. More than one third of NMAs lacked objective criteria to select substances included in their analysis. Despite the lack of standardised criteria for inclusion of molecules in meta-analyses, efforts to minimise potential biases are needed [43,52]. Regardless of what happens in pairwise meta-analyses, drug selection is particularly important for NMAs because differences in the selection of agents influenced the estimates of the network and rankeograms, and results may not reflect the comparative profile of drugs when some treatments are missing [17,53]. The reasons for drug selection should be clearly and explicitly provided in registered protocols, as well as in the methods section of the articles reporting the NMAs.

Almost all NMAs included only randomised controlled trials, with more than 60% using a placebo as the common comparator. Randomised, double-blind, placebo controlled trials are the gold standard to demonstrate the superior efficacy of a new treatment; however, ethical issues about the use of a placebo as a comparator and the possible overestimated effect size of the active compared drug have been discussed [54,55]. Head-to-head trials are increasingly used, as well as observational studies. When carefully designed, the latter can provide critical information about drugs used in the real world and have been recommended for comparative effectiveness research given the few differences between well-designed observational studies and randomised controlled trials [56–58]. In the future, the inclusion of these other types of studies in NMAs will likely increase [59,60]. Although the number of primary studies in NMAs have remained similar over the years, the number of patients included has significantly decreased, probably due to ethical issues and the costs of clinical trials. A study on pairwise meta-analyses showed that only 58.1% (n = 451) reported *a priori* sample size calculations [61]. NMAs typically include more trials than traditional meta-analyses because of multiple comparisons, but sample size calculations are still required. For NMAs, the sample size for a particular treatment comparison should be estimated as the number of patients in a pairwise meta-analysis that provides the same degree and strength of evidence as in the indirect comparison or NMA [62].

The graphical representation of a network (plot) and the description of its geometry offer a visual idea about trials’ sample sizes, tendencies, and available direct and indirect evidence [5,6]. In the future, a standardised way of reporting NMA plots and geometry should be considered as an additional parameter of publication reproducibility. The use of rankograms to display the probability to be the best choice among evaluated treatments is a helpful tool for policy makers [11,18,28]. However, rankograms alone may not be enough, and graphs would depend on both the nature of data used in the NMAs and the statistical method employed [11,63]. The Bayesian approach is the most commonly used, because it provides a straightforward way to make predictions. This model combines the likelihood with a prior probability distribution (which reflects prior belief about possible parameters) to obtain a posterior probability of the parameters, which improves the frequentist approach [1,64]. However, as showed in our results, there is still a lack of reporting the Bayesian based-method in NMAs. Despite widely used, the contrast-based approach, that focus on modeling relative treatment effects,[26,65] was equally poorly described as the recent developed arm-based approach [66,67].

NMAs share other methodological challenges with traditional pairwise meta-analyses (e.g. issues of bias, heterogeneity, and precision) [11]. The statistical strength of NMAs relies on two basic assumptions: consistency and transitivity. An agreement between direct and indirect estimates of a comparison ensures consistency, and a balanced distribution of effect of trials guarantees transitivity [63,68]. However, while heterogeneity and inconsistency are being better addressed in the NMAs, trial-level outliers’ assessment and multivariate meta-analyses are still poorly reported (less than 10% of studies), probably because few research on this field exists [69,70]. To ensure that these aspects are complied with, information on the model characteristics should always be provided in either the main article or the supplementary material. The use of online supplementary material has significantly increased in NMA publications, because it does not increase any cost to publications and can provide important further details [71,72]. This resource should always include a minimum data set of the systematic review (e.g. complete search strategies for at least one database, characteristics of included studies, methodological quality, and risk of bias of included studies) and, when possible, the complete data set with raw data for the NMA (e.g. raw data or single-effect sizes of primary studies for at least the main outcome, software and algorithm/model used, and evaluated statistical parameters).

Many statistical parameters have been properly reported since the first NMA publications, but key aspects such as inconsistency factors, model fit, statistical approach, detection of trial-level outliers, and convergence are still poorly reported. To improve methodological reporting standards, guidelines and statements—such as the recently published PRISMA-NMA extension of 2015—should help researchers to follow similar reporting patterns, enhancing the evidence quality and reproducibility of NMAs [73,74]. Editors and peer reviewers should ensure that authors carefully follow these recommendations, and periodical analyses could identify reporting weaknesses and recommend guideline clarifications.

Our study has some limitations. We included only NMAs of drug interventions, but NMAs of non-pharmacological interventions are also available in the literature; we cannot guarantee that our results are extensive to these other NMAs. Although the quality score tool was created based on the items of internal validity with items of reporting quality to summarize the methodological requirements to perform an NMA, including different items in the score could produce different results. Further studies on methodological quality assessment tools to NMAs should be conducted.

Finally, our map of characteristics of the published NMAs on pharmacological interventions emphasises this tool’s potential as a gold standard method for healthcare evidence synthesis. Publication of NMAs is growing rapidly as a robust tool to make decisions about effectiveness and safety in drug classes. Some weaknesses, like the non-objective drug selection criteria, were identified in the NMA literature that may limit this technique’s credibility and reproducibility.

## Supporting information

S1 Table Complete search strategies. (DOCX)

S2 Table Methodological score. (DOCX)

S1 File PRISMA checklist. (DOC)
