# Supplementary material for: Mapping the characteristics of network meta-analyses on drug therapy: A systematic review
Source: PLoS One. 2018 Apr 30;13(4):e0196644. doi: 10.1371/journal.pone.0196644 (PMC5927429; doi:10.1371/journal.pone.0196644)
Supplement: S1 Table — (DOCX) [file pone.0196644.s001.docx]

| PubMed | ‘Indirect treatment comparison*’ OR ‘Indirect meta-analysis’ OR ‘Indirect meta-analyses’ OR ‘Mixed treatment meta-analysis’ OR ‘Mixed treatment meta-analyses’ OR ‘Multiple treatment meta-analysis’ OR ‘Multiple treatment comparison*’ OR ‘Network meta-analysis’ OR ‘Network meta-analyses’ OR ‘Bayesian meta-analysis’ |
| --- | --- |
| Scopus | ‘Indirect treatment comparison’ OR ‘Indirect treatment comparisons’ OR ‘Indirect meta-analysis’ OR ‘Indirect meta-analyses’ OR ‘Mixed treatment meta-analysis’ OR ‘Mixed treatment meta-analyses’ OR ‘Multiple treatment comparison*’ OR ‘Network meta-analysis’ OR ‘Network meta-analyses’ OR ‘Bayesian meta-analysis’ |

**S1 Table. Complete search strategies**
