# Supplementary material for: Mapping the characteristics of network meta-analyses on drug therapy: A systematic review
Source: PLoS One. 2018 Apr 30;13(4):e0196644. doi: 10.1371/journal.pone.0196644 (PMC5927429; doi:10.1371/journal.pone.0196644)
Supplement: S2 Table — (DOCX) [file pone.0196644.s002.docx]

**S2 Table. Methodological score**

| **Parameter - Systematic review** | **Description** | **Score** |
| --- | --- | --- |
| Drug selection criteria | Criteria for the selection of the drug(s) are stated by the authors. | 1 = objective or non-objective criteria are established  0 = no statement of the reason(s) for choosing the evaluated drugs |
| Search terms | The authors provide in the article (methods section) the main search terms or MeSH terms employed for the electronic searches. | 1 = the search terms are provided in the text  0 = no statement on the search terms employed |
| Search strategies | The authors provide in the article or in online appendix/supplemental material the complete search strategies for at least one database. | 1 = the complete search stragegy is provided and seems reproductible  0 = no statement on the search strategy employed |
| Supplemental material | The authors provide any sort of supplemental material or online appendix (e.g. search strategies, additional analyses, additional results, employed algorithms...). | 1 = at least one supplemental material is provided  0 = no additional material |
| More than two databases | For the systematic review searches, more than two electronic databases (e.g. PubMed and Scopus) were employed. | 1 = at least two electronic databases are employed  0 = less than one database or no statement of the electronic search |
| Manual searches | Besides the electronic databases, the authors declare to search additional sources manually (e.g. Google, Google Scholar). | 1 = manual searches were conducted in at least one source  0 = no manual searches were conducted or no statement of additonal searches |
| Grey literature searches | Besides the electronic databases, the authors declare to search additional non-indexed sources (e.g. abstracts and conferences). | 1 = searches were conducted in grey literature  0 = no manual searches were conducted or no statement of additonal searches |
| PRISMA recommendations | The authors declare to follow PRISMA or PRISMA-NMA statements for the systematic review or present the PRISMA checklist. | 1 = the recommendations were followed  0 = no statement |
| Cochrane recommendations | The authors declare to follow the Cochrane Handbook for Systematic Reviews of Interventions or similar Cochrane guidelines or recommendations. | 1 = the recommendations were followed  0 = no statement |
| PROSPERO register (NHS) | The authors declare to have registered the review in the International Prospective Register of Systematic Reviews and/or present the number of register protocol. | 1 = declaration of the register or number of the protocol  0 = no statement |
| Jadad/Cochrane bias evaluation | Methodological tools of primary studies (Jadad score and/or Cochrane Risk of Bias Tool) were employed in the study for the evaluation of bias. | 1 = at least one of the tools (Jadad score or Cochrane Risk of Bias Tool) was employed  0 = no methodological evaluation was conducted or no statement |
| Provides statistical analyses | The authors describe the statistical analyses (e.g. frequentist and/or Bayesian) in the methods section. | 1 = description of the statistical method  0 = no statement |
| Provides effect size measures | The authors describe the effect size measures (e.g. relative risk, odds ratio, mean difference) in the methods section. | 1 = description of the measures  0 = no statement |
| Provides statistical method | The authors describe the statistical method (e.g. fixed and/or random effect) in the methods section. | 1 = description of the statistical method  0 = no statement |
| Additional statistical analyses | Other analyses (e.g. meta-regression, sensibility analyses, subgroup analyses) are conducted and reported in the methods section as well as in the results and/or supplemental material. | 1 = additional statistical analyses are provided in both the methods and results sections  0 = no additional analyses were conducted or reported |
| Software employed | The software(s) used for statistical analyses is cited by the authors. | 1 = description of the software  0 = no statement |
| Provides inconsistency | The authors describe the methodology for evaluation of NMA inconsistency in the main text or in the supplemental material. | 1 = description of the inconsistency method employed  0 = no statement |
| Provides model fit | The authors describe the methodology for evaluation of model fit of NMA data in the main text or in the supplemental material. | 1 = description of the model fit  0 = no statement |
| Provides convergence | The authors describe the methodology for evaluation of NMA data convergence in the main text or in the supplemental material. | 1 = description of NMA convergence  0 = no statement |
| Provides results from NMA | Results of the NMA are provided in the main text or supplemental material (e.g. matrix of effect size results considering direct and indirect comparisons). | 1 = results are provided for the comparisons  0 = no statement |
| Provides NMA plot | The authors provide the NMA plot (figure) in the main text or in the supplemental material accounting for all the evaluated drugs. | 1 = presence of NMA plot  0 = no statement |
| Provides NMA geometry | The authors describe the NMA geometry accounting for all evaluated drugs and considering at least the node sizes and line thickness. | 1 = description of the NMA geometry  0 = no statement |
| Provides rank order | The authors perform and present (e.g. figure, description in the main text) a rank order for the evaluated drugs considering the results of the NMA resulting in the best therapy, second best, and so on, | 1 = provides a rank order  0 = no statement |
| COI | The authors properly declare any conflict of interest while conducting the study. | -1 = no declaration  0 = declared COI  1 = nothing to declare |
| Financial support | The authors properly declare any financial support (e.g. pharmaceutical industries) while conducting the study. | -1 = no declaration  0 = declared financial support  1 = no financial support |
